# Change of Circulating Vascular Endothelial Growth Factor Level and Reduction of Anhedonia Are Associated in Patients With Major Depressive Disorder Treated With Repetitive Transcranial Magnetic Stimulation

**DOI:** 10.3389/fpsyt.2022.806731

**Published:** 2022-05-31

**Authors:** Monika Elemery, Szilvia Kiss, Peter Dome, Laszlo Pogany, Gabor Faludi, Judit Lazary

**Affiliations:** ^1^János Szentágothai Neuroscience Doctoral School, Semmelweis University, Budapest, Hungary; ^2^National Institute of Mental Health, Neurology and Neurosurgery, Budapest, Hungary; ^3^Department of Psychiatry and Psychotherapy, Semmelweis University, Budapest, Hungary

**Keywords:** anhedonia, antidepressant, brain stimulation, depression, treatment resistant depression

## Abstract

**Aim:**

Vascular endothelial growth factor (VEGF) has been implicated in mediating the effect of antidepressant therapies as it plays a significant role in the neurogenesis. Anhedonia, an endophenotype of major depressive disorder (MDD), is related to the dorsolateral prefrontal cortex, the major focus of brain stimulation in MDD. The aim of our study was to analyze the change of serum VEGF level after rTMS treatment in association with anhedonia.

**Materials and Methods:**

A dataset of 17 patients with TRD who were treated with antidepressants and bilateral rTMS for 2 × 5 days was analyzed. Depression was measured by the Montgomery–Asberg Depression Scale (MADRS) and anhedonia by the Snaith–Hamilton Pleasure Scale (SHAPS) for monitoring the symptom changes. The serum VEGF levels and symptoms were assessed on the first (V1), on the 14th (V2), and on the 28th day (V3). The level of VEGF was measured by ELISA assay.

**Results:**

There was no significant association between MADRS scores and serum VEGF levels at any timepoint. The decrease in the SHAPS score was significantly associated with the increase in VEGF level between V1 and V2 (*p* = 0.001). The VEGF levels were significantly higher in non-responders than in responders (*p* = 0.04). The baseline VEGF level has been proven as a significant predictor of treatment response (*p* = 0.045).

**Conclusion:**

Our results suggest that serum VEGF can be sensitive to the changes of anhedonia during rTMS treatment. Considering that the most widely used depression scales are not applicable for the assessment of anhedonia, measurement of anhedonia in rTMS treatment studies of patients with TRD can be suggested as more appropriate data on distinct pathogenic pathways and specific biomarkers of the disorder.

## Introduction

Major depressive disorder (MDD) is one of the leading disabilities in the world with an account of 7.5% of all years lived with disability according to the WHO report (1). Although there is an increasing body of data on the pathophysiological details underlying the etiology of MDD, which helps in the development of antidepressants as well, the picture is still not complete. Hence, clarifying the molecular pathomechanism and completing the treatment arsenal of MDD are essential goals of neuroscience research.

Besides pharmacotherapeutic and psychotherapeutic methods, repetitive transcranial magnetic stimulation (rTMS) as an alternative therapeutical intervention in the treatment of MDD appeared in the guidelines from the early 2000s (2). The rTMS has been implemented in the international guidelines as a fourth-line treatment in case of treatment-resistant depression (TRD); however, intervention methods are under development and more efficient protocols are appearing in the literature, such as bilateral methods (3). High-frequency stimulation above the left dorsolateral prefrontal cortex (DLPFC) combined with low-frequency stimulation above the right DLPFC region has been proven as an effective treatment for MDD (4). With an improvement of methods and accumulating data on its excellent side effect profile (e.g., its use during pregnancy seems to be more safe and effective than antidepressants), it is predicted that rTMS can step into the first-line treatments in the near future.

Recently, some investigations have been reported on the molecular changes as consequences of rTMS treatment, but we still have insufficient knowledge about these molecular mechanisms. Finding further data on molecular consequences of rTMS as well as identifying the symptom profiles that are sensitive to rTMS are crucial for further treatment optimalization (5).

The neurotrophic and neurogenic hypothesis of MDD suggests that stress-induced mechanisms in the adult brain cause a disturbance in the regulation of growth factors and resulted in a decrease in hippocampus volume linked with symptoms of MDD (6). In line with this, certain antidepressants can reverse this process and it is reported that the mediating effect of the growth factors is essential for their therapeutical effect. The most commonly investigated growth factor is the brain-derived neurotrophic factor (BDNF) and a significant effect of rTMS treatment on BDNF levels was demonstrated (7). The presence and essential role of the vascular endothelial growth factor (VEGF) in the central nervous system have also been proven. This molecule has also been implicated in the neurogenic regulation based on experimental and human data (8), but the effect of rTMS on the level of circulating VEGF has not yet been investigated.

The investigations on the potential association of antidepressant treatment with the levels of circulating VEGF in patients with MDD resulted in conflicting data. Rigal et al. reported that VEGF levels were lower in 469 patients with MDD compared with healthy controls, but it remained unchanged during antidepressant treatment (9). Pisoni et al. analyzed the data of 36 subjects with TRD and they found that baseline VEGF levels were significantly higher in responders than in non-responders; however, the levels were not deviated from those of healthy controls and were not altered due to the antidepressant treatment (10). In another study wherein fluoxetin was administered to patients suffering from MDD, the baseline VEGF levels were significantly lower in healthy controls than in the MDD subgroup, and there were no significant changes in VEGF concentrations after 4 and 8 weeks of follow-up (11). In our earlier published study, we found significantly higher serum VEGF levels in non-responders than in responder patients with MDD treated with antidepressants (12).

Exploration of the neurobiological background of MDD, especially TRD, is also a key point in the prevention of suicidal behavior, as at least 50% of suicidal patients suffer from MDD (13). Suicide is the second leading cause of death among young population and results from suicide research strongly suggest that one of the most important steps of the prevention would be the improvement of treatment of MDD. Our knowledge about the complex neurobiological mechanisms underlying suicidal behavior is intensively extending. Up-to-date data suggest that dysregulations of multiple brain networks contribute to the development of suicidal behavior. Currently, instead of the direct genetically determined suicide risk, the genetic vulnerability for suicide is interpreted within a gene-stress interaction model completed with several other dysfunctional pathways. Besides the conventional neurotransmitter systems (such as serotonergic, noradrenergic, and dopaminergic), the most recent data revealed that the glutamate-glutamine-GABA cycle plays a crucial role in the development of MDD and suicidal behavior (14). Furthermore, the dysfunction of hypothalamic-pituitary-adrenal axis is also related to chronic stress, and it leads to reduced volume of hippocampus and imbalance of inflammatory cytokines. The dysregulation of immune response could be a contributing factor to MDD at risk of suicide, including the VEGF and kynurenine system. The specific role of reduced levels of neurotrophins in impaired neuroplasticity in MDD is also implicated and the potential pathogenic role of VEGF can be considered.

The potential effect of the electroconvulsive therapy (ECT) on the serum levels of VEGF was also investigated in multiple studies. In two studies, baseline peripheral VEGF concentrations were unchanged due to ECT but were significantly higher in responders than in non-responders (15, 16). On the contrary, Ryan et al. reported that VEGF levels increased significantly after ECT (17). Kolshus et al. found that there was no significant change in VEGF gene related miRNAs levels after the ECT intervention, but higher baseline level was measured in patients with psychotic depression (18). Kranaster et al. reported that the VEGF levels in the cerebrospinal fluid were significantly higher in healthy controls compared with patients with TRD, but it was unchanged after ECT (19). However, Ryan et al. have not found any difference in VEGF levels between psychotic and non-psychotic depression (17). Results of a network meta-analysis of 24 studies with 4,190 participants suggested that serum VEGF levels do not differ between healthy and depressed patients (20).

In summary, only a few and heterogenous data exist on the possible changes in levels of peripheral biomarkers due to rTMS treatment of MDD. Differentiating analysis of rTMS’ effect on specific depressive symptoms such as anhedonia would be useful for more precise indication of rTMS intervention.

The aim of this study was to assess the effect of rTMS treatment on the symptoms of MDD and also to test whether there is an association between serum VEGF levels and improvement of the symptoms (included anhedonia assessed with a specific instrument) in patients with MDD after rTMS treatment.

## Materials and Methods

### Subjects

We recruited 17 adult subjects (6 men and 11 women; mean age = 48.5 ± 11.9 years) from a clinical cohort of patients who suffered from treatment-resistant major depression for at least 12 months. The patients were enrolled from the psychiatric department of the Kútvölgyi Clinical Center, Semmelweis University, Budapest. All patients underwent at least two antidepressant trials without adequate clinical response to qualify as treatment resistant. During rTMS treatment, patients continued the previously initiated antidepressant treatment in accordance with international guidelines. The types of medications and their doses administered to participants during the intervention are presented in Table 1. Patients participated voluntarily in the study and agreed to receive rTMS treatment. Before treatment, all patients underwent a detailed clinical evaluation, which included psychiatric, somatic, and neurological examinations. EEG was performed in order to exclude epilepsy or an elevated risk of convulsions. We used a systematic rTMS safety questionnaire for the assessment of potential risk factors (presence of metallic implants; abusive alcohol or benzodiazepine consumption; symptoms of epilepsy, etc.). Exclusion criteria included the presence of any comorbid psychiatric disorder other than MDD and personality disorder, epilepsy, metallic implants, and chronic somatic diseases. Patients with any abnormal parameter in the routine lab test (high or low fasting glucose level, abnormal number of blood cells, elevated level of lipids, abnormal parameters of ions, or liver enzymes, or necroenzymes and low GFR), higher blood pressure than 140/80 mmHg, or abnormal heart frequency and out of normal range of serum TSH concentration were also excluded from the study. Any acute somatic crisis within a half year (included heart attack, stroke, major surgery, massive hemorrhage, sepsis, etc.) was an exclusion criterion, as well as pregnancy or lactation.

**TABLE 1:** Types of different medications and their doses administered to the participants during the rTMS treatment.

Medications	Number of patients	Mean dose of the medications (mg/day)
escitalopram	5	20
fluvoxamine	2	300
venlafaxine	5	165
duloxetine	4	82.5
amitriptylin	1	50
olanzapine	2	3.75
quetiapine	3	41.7
alprazolam	11	0.77
valproate	2	550

### Phenotypic Questionnaires

Depression was assessed by the Montgomery–Asberg Depression Scale (MADRS), which is a worldwide used structured interview for evaluation of depressive symptoms, such as apparent sadness, reported sadness, inner tension, reduced sleep, concentration difficulty, lassitude, inability to feel, pessimistic thoughts, and suicidal thoughts (21). The items are rated on a 0–6 continuum scale (0 = no abnormality, 6 = severe) and the inter-rater reliability between raters has been proven to be good. Although the items of MADRS cover the main general symptoms of MDD, anhedonia cannot be evaluated by this instrument (22). Thus, for a more detailed phenotypic measurement of anhedonia as a core symptom of MDD, we used the Snaith–Hamilton Pleasure Scale (SHAPS) (23), which is a self-rating, 14-item scale with a 4-degree rating scale. The rationale for assessing the anhedonia is also explained by the concept that specific neurophysiological alteration can be responsible for anhedonia. A recently published study reported on the association between decreased resting-state functional connectivity in the dorsal medial prefrontal cortex and anhedonia in patients with MDD (24). Regarding that during the rTMS treatment we stimulate the DLPFC, it can be hypothesized that anhedonia can be reduced effectively by rTMS.

The MADRS and SHAPS scores were recorded at the time of the first visit (V1; pretreatment); at the second visit (2 weeks following the start of rTMS treatment; V2); and at the third visit (4 weeks after the start of rTMS; V3).

### Protocol of Repetitive Transcranial Magnetic Stimulation Treatment

During the rTMS sessions, we used a Magstim Rapid 2 therapy system with the 70 mm air-cooled figure-of-eight-coil. A bilateral method was used with different parameter settings on the two sides (high frequency for the left DLPFC and low frequency on the right side). The localization of treatment was determined according to the Beam F3 method (25) after detection of the motor threshold. The motor threshold was defined as the minimum stimulus intensity necessary to elicit an overt motor response in the contralateral abductor pollicis brevis (APB) or first dorsal interosseus (FDI) muscles. The patients underwent rTMS treatment 5 days a week, and the total number of sessions was ten. The frequency of stimulations over the left DLPFC was 10 Hz, an impulse interval of 4 s, and an intertrain interval (ITI) of 23 s was set (evoking a stimulating effect on cortical neuronal activity). The total number of impulses administered during a session was 2,000. The average duration of rTMS on the left side was 22 min and 30 s. The right side was stimulated continuously, without any interruptions using a frequency of 1 Hz (evoking an inhibitory effect on cortical neuronal activity). On this side of the skull, the total number of the impulses was 990, and the average duration of a session was 16 min and 30 s. We used a side effects questionnaire after each rTMS session, in order to assess the undesired effects, including pain on the skin where the coil was placed, the intensity and duration of headache during treatment, the need for analgesics, otologic side effects, dizziness or nausea during or after the treatment, or any other discomfort related to the rTMS session.

### Assessment of Serum Vascular Endothelial Growth Factor Concentration in the Peripheral Blood

Venous blood samples were collected routinely method at the time of V1, V2, and V3 from patients. All blood sample takings were carried out in the morning before the first eating at the same time point in all patients. Samples were prepared and stored at −80°C until further analysis. Levels of VEGF were quantified using a commercial enzyme-linked immunosorbent assay (ELISA) kit according to the manufacturer’s instructions (Quantikine^®^ human VEGF immunoassay, R&D Systems). The plates were measured on Multiskan EX Microplate Photometer and analyzed by Ascent Software (both from Thermo Electron Corporation, Waltham, MA, United States). Measurements were carried out in duplicate. Results were compared with standard curves.

To reduce the effect of possible factors that can influence the VEGF level, complex clinical (physical examination, including blood-pressure monitoring, height, and body weight) and laboratory evaluations were carried out before enrollment. Patients with elevated levels of fasting blood-glucose (>5.6 mmol/l) or with systolic hypertension (>140 mmHg), higher cholesterol level than the upper level of Adult Treatment Panel III defined borderline hypercholesterolemia (6.2 mmol/l), pregnancy or with marked signs of infection/inflammation were excluded from the study. Major cardiovascular events (acute myocardial infarction, arterial obstructive syndromes of limbs, ischemic stroke) during the half-year period before V1, malignant and/or hematopoietic disorders, and rheumatoid arthritis in the medical history of the patients were also criteria for exclusion.

### Statistical Methods

Comparisons of phenotypic scores and VEGF serum levels between different visit times were performed using repeated-measures ANOVA tests. For the assessment of correlation of changes in serum VEGF concentrations and phenotypic scores, values of V1 were subtracted from the values of V2 and V3 and these delta rests were entered into linear regression models with the enter method. For the comparison of phenotypic measurements, we used Pearson’s correlation test. Differences between categorical variables were analyzed with chi-square test. The predictive value of continuous variable on a binary variable was estimated by the binary logistic regression method. Bonferroni correction has been selected where the software gave this option and *p*-values were accepted as significant if the alpha value was less than 0.05. The statistical computations were performed using SPSS 24.0.

## Results

### Analyses of Montgomery–Asberg Depression Scale and Snaith–Hamilton Pleasure Scale Scores and Serum Vascular Endothelial Growth Factor Levels

The serum VEGF levels of patients were 55.8 ± 35.4 pg/ml at V1, 34.1 ± 23.6 pg/ml at V2, and 45.5 ± 41.6 pg/ml at V3 (Table 2). A repeated-measures ANOVA determined that mean VEGF level did not differ significantly across three time points [*F*(2,32) = 2.91; *p* = 0.07] and *post hoc* test revealed that the decrease between V1 and V2 was not significant (*p* = 0.089). However, mean MADRS score significantly differed across three time points [*F*(2,32) = 6.70; *p* = 0.004]. The results of *post hoc* tests showed that differences between V1 and V2 (*p* = 0.030, 95% CI 0.72–12.34) and V1 and V3 (*p* = 0.007; 95% CI 2.82–14.82) were significant, while the difference between V2 and V3 was not significant (*p* = 0.22; 95% CI −1.51 to 6.10). In the case of SHAPS score, the differences did not show any significance across the three timepoints; however, there was a slight decrease with a trend between V1 and V2 [*F*(2,32) = 2.47; *p* = 0.10; *p*_*v*1–v2_ = 0.067; *p*_*v*2–3_ = 0.69; *p*_*v*1–v3_ = 0.16; Table 2].

**TABLE 2:** Mean values of the serum VEGF levels and the phenotypic scores at the three visits (V1, V2, and V3).

	V1 (Mean ± S.D.)	V2 (Mean ± S.D.)	V3 (Mean ± S.D.)	sig. (V1 vs.V2)	sig. (V2 vs. V3)	sig. (V1 vs. V3)
VEGF (pg/ml)	55.8 ± 32.4	34.1 ± 23.6	45.5 ± 41.6	0.089	0.74	0.71
SHAPS	6.3 ± 4.6	5.0 ± 4.7	5.2 ± 4.8	0.067	0.68	0.16
MADRS	29.5 ± 11.7	22.9 ± 8.3	20.7 ± 12.1	0.030	0.22	0.007

*MADRS, Montgomery–Asberg Depression Scale; VEGF, vascular endothelial growth factor; SHAPS, Snaith–Hamilton Pleasure Scale.*

**FIGURE 1:**
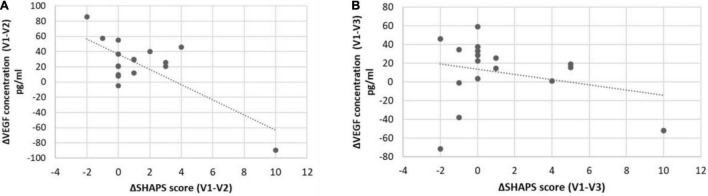
Associations between ΔSHAPS scores and serum ΔVEGF concentrations in responders and non-responders. SHAPS score reduction was significantly (*p* = 0.001; *R* = −0.72) associated with a change in serum VEGF concentration in responders **(A)** but is not significantly (*p* > 0.05; *R* = −0.25) associated in non-responders **(B)**.

For deeper analysis of the potential relationship between VEGF level changes and phenotypic variance alterations, we tested the association of the delta values (differences of VEGF concentration at V1, V2, and V3) with the delta scores (difference of SHAPS and MADRS scores at V1, V2, and V3) by general linear models. According to our analyses, the delta VEGF level was significantly associated with the reduction of the SHAPS score between V1 and V2 (*p* = 0.001; Table 3; Figure 1). Although the mean VEGF level was unchanged in the sample at V2, this association suggested that specifically the SHAPS score reduction was correlated to the elevation of VEGF concentration at V2 compared to V1 and this correlation had a high *R* value (*R* = −0.74; Figure 1) and explained variance (adjusted *R*^2^ = 0.54). In case of the MADRS score, increasing VEGF levels were associated with the attenuation of scores between V2 and V3 with a trend (*p* = 0.07; Table 3).

**TABLE 3:** Correlations between serum VEGF level changes and phenotypic score differences.

	Mean square	*F*	sig.	Adj. *R*^2^
**Δ SHAPS**				
VEGF Δ(V1-V2)	11738	18.4	0.001	0.5
VEGF Δ(V2-V3)	2.3	0.7	0.4	0.02
VEGF Δ(V1-V3)	10.0	1.0	0.3	0.001
**Δ MADRS**				
VEGF Δ(V1-V2)	299.4	2.6	0.1	0.1
VEGF Δ(V2-V3)	181.8	3.9	0.07	0.2
VEGF Δ(V1-V3)	338.6	2.76	0.2	0.09

*Results of linear regression analysis are presented.*

*SHAPS, Snaith–Hamilton Pleasure Scale; VEGF, vascular endothelial growth factor.*

### Comparison of Serum Vascular Endothelial Growth Factor Concentrations in Responder and Non-responder Patients

In the next step, the response to the applied treatments has been determined based on the reduction of symptom scores. Subjects whose MADRS score has been decreased by more than 50% at the V3 were defined as responders [V3_*MADRS*_ < 14.5; (Table 3)]. Non-responders exhibited significantly higher serum VEGF concentrations and SHAPS scores at V1 compared with responders (VEGF_non–responder_ = 69.4 ± 32.5 vs. VEGF_responder_ = 36.6 ± 22.1; *p* = 0.04). The results of binary logistic regression suggested that the serum concentration of VEGF at V1 had a significant predictive value for being responder (*p* = 0.04) and the score of the SHAPS at V1 had a predictive value only with a trend (*p* = 0.06; Table 4).

**TABLE 4:** Results of binary logistic regression analysis of VEGF level and SHAPS scores at V1 on categories of responder or non-responder.

	B	SE	Wald	df	Sig	Exp(B)
VEGF_*V*1_	–1.09	5.46	4.01	1	0.045	0.33
SHAPS_*V*1_	–0.52	2.78	3.57	1	0.059	0.59

*VEGF, vascular endothelial growth factor; SHAPS, Snaith–Hamilton Pleasure Scale.*

## Discussion

We investigated the potential effect of rTMS and antidepressant treatments on serum VEGF levels in 17 patients with TRD. Our results showed that a 14-day rTMS treatment-induced VEGF level is associated with reduced anhedonia score and this relationship accounted for 54% of the explained variance. Furthermore, a higher baseline VEGF level and higher SHAPS score may be a risk factor to be a non-responder to rTMS in our sample. Improvement of MADRS score after 28 days was also associated with an increase in VEGF but only with a trend.

Anhedonia is a special (core) symptom characterized by loss of pleasure, loss of interest in previously enjoyed activities, and pessimism. It can be regarded as a transdiagnostic endophenotype of negative symptoms of schizophrenia, MDDs, and other specific psychiatric disorders (26), which is associated with the dysfunction of specific brain regions, namely, the DLPFC. That is the reason why measurement of anhedonia before and after the brain stimulation has been strongly suggested by Spano et al. They concluded that anhedonia is an underexplored condition in neuromodulation trials, and they suggested that anhedonia can be a valuable transdiagnostic dimension that requires further examination in order to discover new clinical predictors for treatment response (26). In line with this, Siddiqi et al. analyzed circuit maps in association with distinct phenotypes of 14 rTMS clinical trials and they concluded that anhedonia responded best to stimulation of left DLPFC, while the so-called anxiosomatic biotype was associated with the dorsomedial prefrontal cortex (27). Further, Light et al. demonstrated the effectiveness of the rTMS treatment in the reduction of anhedonia measured not only with a psychometric scale but also with a special “happy face” task in a sample of patients with MDD (28).

Relevance of brain stimulation in the attenuation of anhedonia can be explained by the results of Rzepa et al. on the decreased resting-state functional connectivity in the dorsal medial prefrontal cortex in association with anhedonia of 86 patients with MDD (24). Further investigations are needed to how to maintain this effect of rTMS on anhedonia.

On the contrary, the significant pathological role of growth factors in the development of anhedonia has been implicated. One of the conceptual frameworks, anhedonia may be regarded as an adaptive response to the repetitive stress induced neuronal microinjury to prevent patients from engaging in activities that require excessive effort (29–31). In animal experiments, anhedonic-like behaviors were related to changes in BDNF metabolism (32), but there are only a few clinical studies focused so far on the association between BDNF and anhedonia. Wu et al. reported that the increased ratio of mature BDNF to precursor BDNF was found in patients with major depressive disorder with severe anhedonia measured by the same questionnaire that we used in our presented study (SHAPS) (33). Another interesting result showed that the fibroblast growth factor (FGF22) was also associated with anhedonia (34). Along this line, the potential similar role of other growth factors in the CNS can be assumed, such as the VEGF.

The majority of data on serum VEGF level differences between healthy controls and patients with MDD or TRD showed that a lower concentration is present in patients (nevertheless, the meta-analysis of these data suggested no significant difference). However, by testing the potential associations, it was revealed that delta VEGF concentrations did not show any correlations with changes in the MADRS score between V1 and V3, only in responders. Our results suggest that the baseline VEGF level is a significant predictor of treatment response to rTMS, which is in line with our previous results on the association between baseline serum VEGF level and treatment response to pharmacotherapy (12). Comparing responder and non-responder patients with MDD to treatment with rTMS, a higher baseline anhedonia score was found in non-responders (35). In our investigation although the baseline anhedonia score was higher in the group of non-responders, this difference has not been proven as significant, maybe due to the small size of the sample. The lack of association between MADRS score change and VEGF level change suggests that effective treatment of anhedonia as a core symptom of antidepressant resistant depression can be dependent on the capacity of the VEGF pathway and stimulation of the DLPFC in patients with MDD. It can be recommended that assessment of baseline serum VEGF level and anhedonia score can be included in the preparation process of rTMS treatment planning procedure and patients with higher VEGF level and anhedonia score can receive more intensive brain stimulation.

Our results on the significant reduction of MADRS score during rTMS treatment are partly in contrast with a currently published large-scale meta-analysis of rTMS results on sham-controlled data. Homan et al. reported an analysis of 130 randomized controlled studies, which included results of 5,748 patients with psychiatric conditions. They found that only a minimal increase in variability can be observed after active stimulation compared with sham that did not reach statistical significance (3). Although in our study sham control was not applied, our results can suggest that rTMS can improve specifically anhedonia *via* increase in VEGF concentration. In other words, RCT studies on rTMS treatment outcomes can be improved by rating specific symptom profiles and serum biomarkers together.

Our study has some limitations. First, the size of the sample treated with rTMS was small and we had no possibility for a sham-controlled comparison of data. Further investigations are required for confirmation of association between serum VEGF level as a biomarker of treatment effectiveness in MDD. Further studies with sham-controlled design, larger sample size, and the use of different add-on antidepressants are needed.

## Conclusion

Our results confirmed the effectiveness of rTMS combined with antidepressants in the treatment of TRD. Serum VEGF level changes were not associated with depressive symptom improvement due to rTMS, but it is strongly correlated with reduction of anhedonia. Our results suggest that impaired neuroplasticity contributes specifically to the development of anhedonia, and it can be repaired by rTMS interventions successfully. Regarding that the MADRS scale does not measure anhedonia, special instruments such as the SHAPS can be recommended to be used in scientific research to measure effects of rTMS on affective symptoms. A significant predictive value of baseline serum VEGF level for treatment response indicated that the inducibility of neurotrophic system is an essential state factor of treatment outcome, especially the attenuation of anhedonia by the rTMS treatment.

## Data Availability Statement

The raw data supporting the conclusions of this article will be made available by the authors, without undue reservation.

## Ethics Statement

The studies involving human participants were reviewed and approved by Hungarian Central Ethics Committee (8491-9/2018/EÜIG). The patients/participants provided their written informed consent to participate in this study.

## Author Contributions

ME contributed to the enrollment of patients, performance of the rTMS treatment and sample collection, and preparation of the manuscript draft. SK participated in the preparation of the rTMS treatment and management of the data set. PD contributed to the design of the clinical protocol of rTMS treatment and the preparation of the manuscript draft. LP contributed to patient enrollment and preparation of the manuscript draft. GF contributed to study design and reviewed the manuscript. JL contributed to study design, organizing the assays of serum VEGF concentration, statistical analyses, and final approval of the work. All authors contributed to the article and approved the submitted version.

## Conflict of Interest

The authors declare that the research was conducted in the absence of any commercial or financial relationships that could be construed as a potential conflict of interest.

## Publisher’s Note

All claims expressed in this article are solely those of the authors and do not necessarily represent those of their affiliated organizations, or those of the publisher, the editors and the reviewers. Any product that may be evaluated in this article, or claim that may be made by its manufacturer, is not guaranteed or endorsed by the publisher.
